# Matching proposed clinical and MRI criteria of aggressive multiple sclerosis to serum and cerebrospinal fluid markers of neuroaxonal and glial injury

**DOI:** 10.1007/s00415-024-12299-z

**Published:** 2024-03-27

**Authors:** Martin A. Schaller-Paule, Michelle Maiworm, Jan Hendrik Schäfer, Lucie Friedauer, Elke Hattingen, Katharina Johanna Wenger, Frank Weber, Jasmin Jakob, Falk Steffen, Stefan Bittner, Yavor Yalachkov, Christian Foerch

**Affiliations:** 1 Department of Neurology, University Hospital Frankfurt, Goethe University Frankfurt, Schleusenweg 2-16, 60528 Frankfurt, Germany; 2 Practice for Neurology and Psychiatry Eltville, 65343 Eltville, Germany; 3 Institute of Neuroradiology, University Hospital Frankfurt, Goethe University Frankfurt, Frankfurt, Germany; 4 Neurological Clinic Cham, Cham, Germany; 5 Department of Neurology, Focus Program Translational Neuroscience (FTN) and Immunotherapy (FZI), Rhine-Main Neuroscience Network (rmn2), University Medical Center of the Johannes Gutenberg University Mainz, Mainz, Germany

**Keywords:** Biomarkers, Aggressive disease, Multiple sclerosis, Neurofilament light chain, Prognosis, Disease activity

## Abstract

**Background:**

Definitions of aggressive MS employ clinical and MR imaging criteria to identify highly active, rapidly progressing disease courses. However, the degree of overlap between clinical and radiological parameters and biochemical markers of CNS injury is not fully understood. Aim of this cross-sectional study was to match clinical and MR imaging hallmarks of aggressive MS to serum/CSF markers of neuroaxonal and astroglial injury (neurofilament light chain (sNfL, cNfL), and glial fibrillary acidic protein (sGFAP, cGFAP)).

**Methods:**

We recruited 77 patients with relapsing–remitting MS (RRMS) and 22 patients with clinically isolated syndrome. NfL and GFAP levels in serum and CSF were assessed using a single-molecule-array HD-1-analyzer. A general linear model with each biomarker as a dependent variable was computed. Clinical and imaging criteria of aggressive MS, as recently proposed by the ECTRIMS Consensus Group, were modeled as independent variables. Other demographic, clinical or laboratory parameters, were modeled as covariates. Analyses were repeated in a homogenous subgroup, consisting only of newly diagnosed, treatment-naïve RRMS patients presenting with an acute relapse.

**Results:**

After adjusting for covariates and multiplicity of testing, sNfL and cNfL concentrations were strongly associated with the presence of ≥2 gadolinium-enhancing lesions (*p*_sNfL_ = 0.00008; p_cNfL_ = 0.004) as well as the presence of infratentorial lesions on MRI (*p*_sNfL_ = 0.0003; p_cNfL_ < 0.004). No other clinical and imaging criteria of aggressive MS correlated significantly with NfL or GFAP in serum and CSF. In the more homogeneous subgroup, sNfL still was associated with the presence of ≥2 gadolinium-enhancing lesions (*p*_sNfL_ = 0.001), presence of more than 20 T2-lesions (*p*_sNfL_ = 0.049) as well as the presence of infratentorial lesions on MRI (*p*_sNfL_ = 0.034), while cNfL was associated with the presence of ≥2 gadolinium-enhancing lesions (*p*_sNfL_ = 0.011) and presence of more than 20 T2-lesions (*p*_sNfL_ = 0.029).

**Conclusions:**

Among proposed risk factors for an aggressive disease course, MRI findings but not clinical characteristics correlated with sNfL and cNfL as a marker of neuroaxonal injury and should be given appropriate weight considering MS prognosis and therapy. No significant correlation was detected for GFAP alone.

## Introduction

Over the past decades, the phenotypes of relapsing–remitting and primary progressive multiple sclerosis (MS) have been intensively studied [1]. More recently, several attempts have been made to identify a subgroup of patients with a particularly aggressive disease course and a rapid accumulation of significant disability [2]. Early access to high efficacy therapies can slow down disease progression and prevent severe long-term disability accumulation [2]. Therefore, tools to early identify those patients at risk are urgently needed.

Clinical parameters of disease activity such as number and severity of relapses, Expanded Disability Status Scale (EDSS) deterioration, or the achievement of EDSS milestones as well as treatment failure have been proposed as markers for aggressive disease [2]. Increasingly, MRI activity markers such as new or enlarging T2-hyperintense lesions or the presence of gadolinium-enhancing lesions (Gd+) are integrated in defining an aggressive disease course [2]. However, a major drawback of commonly applied definitions of an aggressive MS course is the need for either retrospective assessment or a long-term prospective observation, delaying the identification of aggressive disease courses and, therefore, the initiation of highly effective therapies [2].

Recently, the European Committee for Treatment and Research in Multiple Sclerosis (ECTRIMS) consensus group highlighted clinical and paraclinical parameters (e.g., symptoms at first relapse, relapse severity and recovery, EDSS ≥ 3 in the first year, presence of spinal cord and infratentorial lesions, gadolinium-enhancing lesions as well as lesion load) as likely relevant for the identification of aggressive MS [2]. Still, the extent to which these conventional markers are matched to the degree of neuroaxonal and astroglial damage remains unclear.

On a pathophysiological level, an aggressive MS disease course would be associated with more pronounced neuroinflammation and neurodegeneration and thus with higher serum/CSF concentrations of neuroaxonal and astroglial injury markers. Serum/CSF concentrations of neurofilament light chain (NfL) and glial fibrillary acidic protein (GFAP) increase subsequently to neuroaxonal or astroglial damage [3, 4] and are, therefore, strong candidates as biochemical markers of aggressive MS. The correlation of clinical and MRI hallmarks of increased MS disease activity with biochemical markers of CNS injury could facilitate in the process of establishing defining criteria of an aggressive MS disease course, and provide pathophysiological validation.

Aim of this study was to investigate the association between hallmarks of aggressive MS disease activity, as defined by the ECTRIMS Consensus Group, and markers of neuroaxonal and astroglial injury in the serum and CSF in people with MS (pwMS). In addition, pwMS with defined elevated values for serum NfL and serum GFAP as well as the combination of both are characterized in order to better understand the predictive value of both biomarkers.

## Methods

### Study population

Patients were recruited between October 2017 and December 2020 at the Department of Neurology, University Hospital Frankfurt, where they were referred to due to suspected MS based either on a clinical observation or based on MRI imaging results or, if they already had an established MS diagnosis, due to a novel clinical/imaging finding. Subjects were included in the study if they were ≥18 years old, agreed to participate, were scheduled for a clinically indicated lumbar puncture and the diagnostic workup resulted in the diagnosis of a relapsing–remitting multiple sclerosis (RRMS) or clinically isolated syndrome (CIS) according to the 2017 revision of the McDonald criteria [5]. The study was performed in accordance with the Code of Ethics of the World Medical Association (Declaration of Helsinki) for experiments involving humans and was approved by the local ethics committee at the University Hospital Frankfurt. Written informed consent was obtained from all subjects. All subjects underwent a neurological examination, laboratory tests, lumbar puncture, and MR-imaging as part of the clinical routine. Serum and CSF samples were obtained during a clinically scheduled sample collection. Degree of physical disability was evaluated by a neurologist with the Kurtzke EDSS [6]. MR-imaging data were examined by specialized neuroradiologists (M.H. and K. W.) (Table 1).

**Table 1:** Potential red flags and parameters of aggressive disease

Probable red flags^a^	Potential parameters^b^
Motor symptoms at onset [7, 8]	Male sex [9, 10]
Age > 35 years at symptom onset	Older age at onset (>35 or >40 years) [10–12]
EDSS ≥ 3.0 in the first year	Severe relapses of ≥1 point increase in EDSS or ≥2 points in any functional system
Presence of pyramidal signs in the first year of disease evolution [12]	Affecting motor, cerebellar, cognition, or sphincter functions [11, 13–17]
≥2 Gd+ lesions at the time of disease onset [18, 19]	High relapse frequency (≥3) within the first 2 years after MS onset [20]
High T2 lesion load [21, 22] [0, 1–3, 4–9, ≥10, ≥20 T2 lesions]	Poor recovery from the first two relapses [7]
	Presence of infratentorial lesions [23, 24]
	Presence of ≥1 spinal cord lesion, symptomatic or asymptomatic [23, 25, 26]
	Elevated IgG Index [27] [oligoclonal bands]
	If a relapse has led to a severe deficit relevant to everyday life after exhaustion of relapse therapy

^a^ With additional research and validation, one or more of the following characteristics, along with physician judgement, may be considered a red flag for poor short- and long-term prognosis and thus could imply initiation of a highly effective therapy

^b^ Potential parameters associated with more aggressive disease course (anyone or a combination of these characteristics may signal aggressive disease, and all are deserving of further exploration and if possible, validation in the context of assessing severity of disease and poor prognosis)

### Serum and CSF measurements

Blood tubes containing coagulation activating agent (S-Monovette, 4.7 ml, Sarstedt) as well as CSF tubes (Greiner PS, 14 ml) were used for blood/CSF sample collection. Only CSF samples with erythrocyte count below 500/μl were included. Samples were centrifuged at 3000 rpm for 10 min, pipetted and frozen within 60 min of collection at −20 °C according to an established procedure reported in previous studies from our and other working groups [28–35]. Every 4 weeks the collected samples were moved to a −80 °C freezer, where they remained frozen until the final laboratory measurements. The serum and CSF samples were sent to the Department of Neurology at the University of Mainz for NfL and GFAP quantification. All laboratory technicians performing the measurements were blinded to the clinical data. The Single Molecule Array (SIMOA) HD-1 analyzer (Quanterix^®^) was utilized to determine NfL and GFAP levels in serum and CSF using the Neurology 4-Plex A Advantage Kit (Quanterix^®^) according to the manufacturer’s instructions. The NfL and GFAP values were measured with two different kit LOTs. The inter-LOT Coefficient of Variability (CV) for the two control samples with high and low concentration was below 10%. In CSF, manually counted cell counts for leukocytes and erythrocytes per mm^3^, CSF/serum albumin quotient (Qalb), and intrathecal Immunoglobulin G (IgG) synthesis were assessed. The Qalb as a marker of Blood–brain barrier dysfunction was interpreted based on the age-adjusted upper reference limit as introduced by Reiber et al. (Qalb = 4 + Age/15) [36].

### Magnetic resonance imaging (MRI)

MRI was performed in clinical routine with acquisition of at least a sagittal 3D double inversion recovery (DIR), a 2D T2-weighted as well as 2D and 3D fluid-attenuated inversion recovery (FLAIR) sequence. Furthermore, a gadolinium-contrast enhanced T1-weighted sequence was assessed. Further adjustments to the MRI protocol were done according to the respective clinical indication. MRI field strength varied between 1.5 and 3 Tesla. T2-lesion count was assessed by two experienced neuroradiologists (M.H. and K.W.). Lesion volume was assessed semi-automatically using 3D sequences [29]. In total, MRI of the brain was available in 94 patients (95%) and spinal cord MRI was available in 55 patients (55%).

### Statistical analysis

One-way analysis of variance (ANOVA), Spearman rank correlations, non-parametric Mann–Whitney *U* tests and Pearson’s Chi-squared tests with significance level set at *p* < 0.05 were used to identify associations between NfL in serum (sNfL) and CSF (cNfL), as well as GFAP in serum (sGFAP) and CSF (cGFAP), with baseline demographic, clinical, imaging and laboratory characteristics such as age, sex, disease phenotype, body-mass index (BMI), EDSS, gadolinium-enhancing lesions (Gd+), presence of acute relapse, blood-CSF barrier dysfunction, CSF leukocytes count, intrathecal synthesis of immunoglobulin G (IgG), positive oligoclonal bands and CSF/blood albumin quotient. Baseline characteristics with significant association were later modeled as covariates in the general linear model (GLM) for the respective biomarker. In the primary analysis, for each of the potential predictors of aggressive disease course (Table 3, 4) four GLMs with each of the biomarkers sNfL, cNfL, sGFAP and cGFAP as a dependent variable were computed, while controlling for the baseline characteristics. Before entering the analysis, serum and CSF parameters were log-transformed. Results were corrected for false-discovery rate (FDR) using the Benjamini–Hochberg procedure. Adjusted and corrected for multiple comparisons, *q* values for each predictor were calculated and significance level was set at *q* < 0.05. In an exploratory, secondary analysis, based on recent literature, pwMS with sNfL > 10 pg/ml [37], sNfL in the 4th quartile (>17.5 pg/ml), sGFAP in the 4th quartile (>109 pg/ml) as well as patients with combined elevation of sNfL and sGFAP in the 4th quartile [38] were additionally assessed and characterized in regard to baseline parameters and presence of the ECTRIMS criteria for an aggressive disease course (Table 5, 6).

To explore if our results would be confirmed if the analysis is restricted only to a more homogenous subgroup, we compared sNfL, sGFAP, cNfL, cGFAP and z-scores for sNfL computed based on the references suggested by Benkert et al. [39] for RRMS groups defined by the factors identified as significant predictors in the initial analysis (presence of at least 2 Gd+ lesions; presence of infratentorial lesions; presence of >20 T2 lesions in brain MRI). This analysis was computed using independent samples t-tests with Levene’s tests for equality of variances and included only a homogeneous subgroup of *n* = 59 newly diagnosed, treatment-naïve RRMS patients without any previous disease-modifying treatment, presenting with an acute relapse and available samples for all four biomarkers sNfL, cNfL, sGFAP, cGFAP.

## Results

### Patient characteristics

In total 99 Patients with relapsing–remitting multiple sclerosis (RRMS; *n* = 77) or clinically isolated syndrome (CIS; *n* = 22) according to the revised McDonald criteria from 2017 [5] were included in the study. The patient characteristics were: 77/99 female (78%) and 22/99 male patients (22%) with mean age of 34 years (SD ± 9.5 years) and mean EDSS of 1.9 (SD ± 1.2). Out of the total 99 patients in this study, 95 patients were first diagnosed with either RRMS or CIS, 84 of whom having recently suffered an acute relapse, and 11 patients that underwent lumbar punction solely because of recent MRI abnormalities without an acute clinical correlate. The remaining 4 patients were already under disease-modifying therapy with previous diagnosis of RRMS (2 patients under natalizumab, 1 under ocrelizumab, 1 under fingolimod) and received lumbar puncture either to rule out progressive multifocal leukoencephalopathy (PML, *n* = 2) or because of an atypical clinical presentation (*n* = 2). Time between relapse onset and assessment of blood samples was less than 6 weeks, in most patients within 2 weeks after relapse onset. When necessary, relapses were treated with intravenous methylprednisolone but only after blood and CSF samples were taken. Time between sample acquisition and MRI image acquisition was 4.9 days. Demographic, clinical, and laboratory characteristics and their respective association with serum/CSF biomarker concentrations are displayed in Table 2.

**Table 2:** Demographic, clinical, and laboratory characteristics of the sample in relation to serum and CSF biomarkers

	Patient sample *n* = 99	Serum NfL	CSF NfL	Serum GFAP	CSF GFAP
*Baseline data*					
Age (years), mean (SD)	34.0 (±9.5)	*r* = −0.004 *p* = 0.97^b^	*r* = −0.066 *p* = 0.52^b^	*r* = −0.017 *p* = 0.87^b^	*r* = 0.127 *p* = 0.21^b^
Female, *n* (%)	77 (77.7)	0.16^c^	0.55^c^	0.13^c^	0.08^c^
Male, *n* (%)	22 (22.2)				
RRMS, *n* (%)	77 (77.7)	***p* = 0.001**^c,1^ ***U* = 424**	***p* = 0.001**^c,1^ ***U* = 454**	0.09^c^	0.54^c^
CIS, *n* (%)	22 (22.2)				
BMI (kg/m^2^), mean (SD)	25.0 (±5.2)	*r* = −0.03 *p* = 0.78^b^	*r* = 0.09 *p* = 0.40^b^	***r* = **−**0.23**^2^ ***p* = 0.03**^b^	*r* = −0.12 *p* = 0.28^b^
*Clinical and laboratory data*					
EDSS, mean (SD)	1.9 (±1.2)	*r* = 0.08 *p* = 0.45^b^	*r* = 0.03 *p* = 0.75^b^	***r* = 0.21**^3^ ***p* = 0.04**^b^	*r* = 0.07 *p* = 0.48^b^
Gadolinium-enhancing T1-weighted lesions on MRI, *n* (%)					
Yes	58 (58.6)	***p* = 0.041**^a^ **p**_**LSD**_** = 0.017**^4^	0.101^a^	0.87^a^	0.44^a^
No	35 (35.4)				
Gd not administered	6 (6.1)				
With acute relapse, *n* (%)	86 (86.9)	0.23^c^	0.42^c^	***p* = 0.02**^c,5^ ***U* = 661**	0.92^c^
Blood-CSF barrier dysfunction, *n* (%)	19 (19.2)	0.76^c^	0.69^c^	0.29^c^	0.37^c^
CSF leukocytes count (mm^−3^), mean (SD)	9.9 (±12.6)	***r* = 0.29**^6^ ***p* = 0.004**^b^	***r* = 0.36**^6^ ***p* < 0.0001**^b^	*r* = −0.03 *p* = 0.75^b^	*r* = −0.07 *p* = 0.49^b^
Intrathecal synthesis of IgG, *n* (%)	60 (60.6)	0.15^c^	0.33^c^	0.64^c^	0.27^c^
Positive oligoclonal bands, *n* (%)	90 (90.9)	***p* = 0.034**^c,7^ ***U* = 560**	0.11^c^	0.19^c^	0.71^c^
CSF/blood albumin quotient, mean (SD)	5.0 (±2.1)	*r* = 0.02 *p* = 0.86^b^	*r* = 0.19 *p* = 0.06^b^	*r* = −0.06 *p* = 0.55^b^	***r* = 0.21**^8^ ***p* = 0.04**^b^

*RRMS* relapsing–remitting multiple sclerosis, *CIS* clinically isolated syndrome, *EDSS* Expanded Disability Status Scale, *Gd* gadolinium, *MRI* magnetic resonance imaging, *CSF* cerebrospinal fluid, *IgG* immunoglobulin G, *NfL* neurofilament light, *GFAP* glial fibrillary acidic protein, SD standard deviation

The patients were compared with regard to their demographic, clinical and laboratory characteristics using one-way analysis of variance (ANOVA, ^a^) as well as Spearman rank correlation (^b^) and Chi-square tests (^c^), if not indicated otherwise, two-sided. Significance level was set at *p* < 0.05 (bold). Non-significant p values are provided for every biomarker without further data

^1^ Serum and CSF NfL values were higher in RRMS patients compared to CIS patients

^2^ Serum GFAP values decrease with higher BMI

^3^ Serum GFAP increases with higher EDSS

^4^ Higher Serum NfL values were find in patients with Gd-enhancing lesions, post-hoc Fisher’s Least Significant Difference (LSD) analysis *p* value is provided in brackets between the “yes” and the “no” subgroup

^5^ Serum GFAP values were higher in patients with acute relapse

^6^ Serum and CSF NfL showed a positive correlation with CSF leukocytes count

^7^ Serum Nfl values were higher when positive oligoclonal bands were found

^8^ CSF GFAP decreases with higher CSF/blood albumin quotient

### Markers of neuroaxonal and astroglial injury

For age and biological sex, no significant correlations were found for all the assessed biomarkers. Higher NfL concentration in serum was significantly associated with the disease phenotype (*p* = 0.001, *U* = 424), with higher concentrations in RRMS-patients (median 12.32 pg/ml, IQR 12.71 pg/ml) than in CIS-patients (median 6.97 pg/ml, IQR 6.18 pg/ml). Moreover, sNfL concentration increased with CSF leukocyte count (*r* = 0.29, *p* = 0.004) and was higher in patients with positive oligoclonal bands (*p* = 0.034, *U* = 560; median 11.69 pg/ml with IQR 11.27 pg/ml vs. 8.39 pg/ml with IQR 6.89 pg/ml). The sNfL concentration was significantly higher in patients with gadolinium-enhancing lesions than in those without (*p* = 0.041, median 12.95 pg/ml with IQR 14.83 pg/ml vs. 8.72 pg/ml with IQR 6.87 pg/ml).

Disease phenotype also was significantly associated with higher CSF NfL concentrations (*p* = 0.001), with higher concentrations in RRMS (median 2082 pg/ml, IQR 2652.07 pg/ml) than in CIS patients (median 1025.37 pg/ml, IQR 1141.43 pg/ml). Furthermore, CSF NfL showed a significant positive correlation with CSF leukocytes count (*r* = 0.36, *p* < 0.0001).

Serum GFAP showed a negative correlation with BMI (*r* = −0.23, *p* = 0.03). A positive correlation between sGFAP and EDSS (*r* = 0.21, *p* = 0.04) was found. Higher serum GFAP concentration was significantly associated with the presence of an acute relapse (*p* = 0.02, median 83.01 pg/ml with IQR 50.14 pg/ml vs. 8.75 pg/ml with IQR 8.86 pg/ml). For cGFAP, a negative correlation with CSF/blood albumin quotient (*r* = −0.06, *p* = 0.04) was found.

### Matching biomarker concentrations to criteria of an aggressive MS disease course

Increased sNfL concentrations were strongly associated with the presence of ≥2 Gd+ lesions (*p* = 0.00008; *q* = 0.0035) as well as with the presence of infratentorial lesions (*p* = 0.0003; *q* = 0.0065), after adjusting for those covariates found significant in the primary analysis (see Table 1). The presence of 20 or more T2 lesions in brain MRI was also associated with increased serum NfL (*p* = 0.018), but this result was rendered insignificant by the correction for multiple comparisons (*q* = 0.16). These associations were also found in treatment naïve patients with relapsing–remitting MS suffering acute relapse with higher sNfL levels in patients with at least 2 Gd+ lesions (*p* = 0.001, 1.27 ± 0.27 pg/ml vs. 1.04 ± 0.21 pg/ml) as well as in patients with infratentorial lesions (*p* = 0.034; 1.24 ± 0.26 pg/ml vs. 1.08 ± 0.26 pg/ml) and with 20 T2-lesions or more in brain MRI (*p* = 0.049; 1.26 ± 0.25 pg/ml vs. 1.11 ± 0.27 pg/ml). Furthermore, sNfL z-scores in the RRMS subgroup were found to be significantly increased in patients with at least 2 gadolinium enhancing lesions (*p* = 0.013; 2.33 ± 0.88 vs. 1.62 ± 1.07) and in patients with infratentorial lesions (*p* = 0.03; 2.26 ± 0.89 vs. 1.67 ± 1.12).

**Fig. 1:**
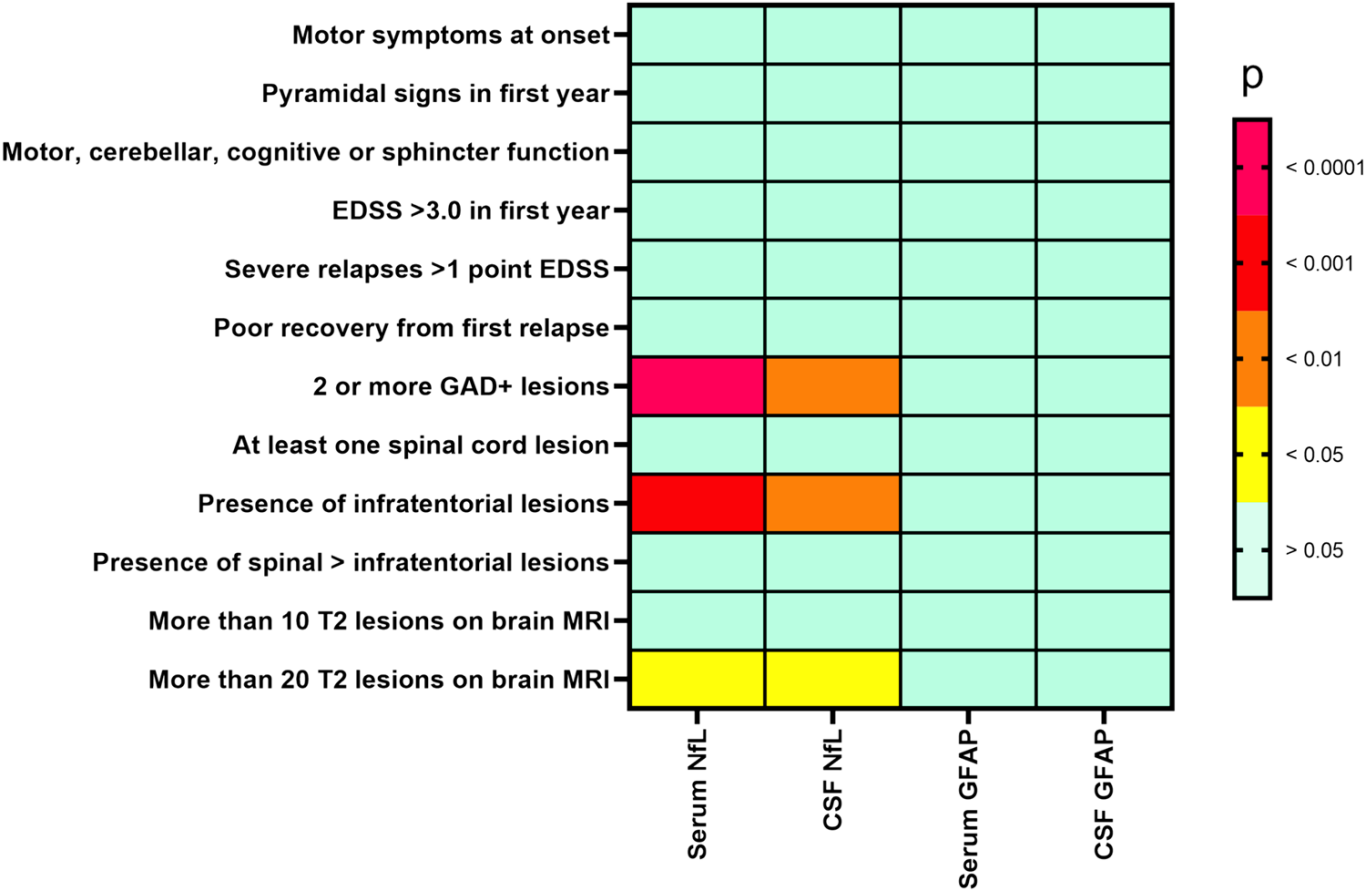
Heat plot. This heat plot diagram shows potential clinical and imaging predictors of aggressive MS disease course (lines) with NfL and GFAP levels in serum and CSF (columns) analyzed in this study. Colors indicate the results of the general linear model provided as p values with the respective biomarker as a dependent variable, while controlling for the baseline characteristics. It is apparent that only imaging parameters correlate with NfL levels in serum and CSF, while no significant correlation was found for clinical parameters or for GFAP. EDSS = Expanded Disability Status Scale; Gd+ MRI = magnetic resonance imaging with gadolinium administration; CSF = cerebrospinal fluid; IgG = immunoglobulin G; NfL = neurofilament light; GFAP = glial fibrillary acidic protein

The cNfL concentration showed significant correlations with presence of ≥2 Gd+ lesions (*p* = 0.004; *q* = 0.048) as well as with the presence of infratentorial lesions (*p* < 0.004; *q* = 0.048). Presence of ≥20 T2 lesions in brain MRI showed a correlation with cNfL levels (*p* = 0.019), which did not remain significant after correcting for multiple comparisons (*q* = 0.15). After controlling for covariates and correction for multiple comparisons, none of the clinical and paraclinical markers for aggressive MS could be predicted either with sGFAP or cGFAP. In the smaller subgroup, increased cNfL was strongly associated with the presence of ≥2 Gd+ lesions (*p* = 0.011, 3.52 ± 0.42 pg/ml vs. 3.25 ± 0.35 pg/ml) as well as presence of at least 20 T2-brain lesions (*p* = 0.029; 3.55 ± 0.36 pg/ml vs. 3.31 ± 0.40 pg/ml) (Table 3, 4; Fig. 1).

**Table 3:** Clinicoradiological markers of aggressive MS disease and NfL

	Serum NfL		CSF NfL	
	*p*	*q* (FDR)	*p*	*q* (FDR)
Motor symptoms at onset	0.476	n.s	0.378	n.s
Pyramidal signs in first year	0.355	n.s	0.335	n.s
Motor, cerebellar, cognitive or sphincter function	0.464	n.s	0.727	n.s
EDSS > 3 in first year	0.815	n.s	0.262	n.s
Severe relapses > 1 point EDSS	0.849	n.s	0.928	n.s
Poor recovery from first relapse	0.91	n.s	0.77	n.s
2 or more Gd+ lesions	**0.00008**	**0.0035**	**0.004**	**0.048**
At least one spinal cord lesion	0.295	n.s	0.158	n.s
Presence of infratentorial lesions	**0.0003**	**0.0065**	**0.004**	**0.048**
Presence of spinal > infratentorial lesions	0.051	n.s	0.108	n.s
More than 10 T2 lesions on brain MRI	0.214	n.s	0.529	n.s
More than 20 T2 lesions on brain MRI	**0.018**	0.16	**0.019**	0.15

*EDSS* Expanded Disability Status Scale, *Gd*+ gadolinium-enhancing lesion, *NfL* neurofilament light chain, *FDR* false discovery rate, *n.s.* not significant

Potential clinical and imaging predictors of aggressive MS disease course computed with the biomarkers sNfL and cNfL as a dependent variable, while controlling for the baseline characteristics. Before analysis, serum and CSF parameters were log-transformed. Results were corrected for false-discovery rate (FDR) using the Benjamini–Hochberg procedure. Adjusted and corrected for multiple comparisons *q* values for each predictor were calculated and significance level was set at *q* < 0.05. Significant results are highlighted in bold

**Table 4:** Clinicoradiological markers of aggressive MS disease and GFAP

	Serum GFAP		CSF GFAP	
	*p*	*q* (FDR)	*p*	*q* (FDR)
Motor symptoms at onset	0.890	n.s	0.240	n.s
Pyramidal signs in first year	0.200	n.s	0.185	n.s
Motor, cerebellar, cognitive or sphincter function	0.666	n.s	0.533	n.s
EDSS > 3 in first year	0.552	n.s	0.122	n.s
Severe relapses > 1 point EDSS	0.571	n.s	0.461	n.s
Poor recovery from first relapse	0.509	n.s	0.101	n.s
2 or more Gd+ lesions	0.877	n.s	0.760	n.s
At least one spinal cord lesion	0.326	n.s	0.712	n.s
Presence of infratentorial lesions	0.194	n.s	0.587	n.s
Presence of spinal > infratentorial lesions	0.961	n.s	0.099	n.s
More than 10 T2 lesions on brain MRI	0.775	n.s	0.856	n.s
More than 20 T2 lesions on brain MRI	0.556	n.s	0.761	n.s

*EDSS* Expanded Disability Status Scale, *Gd+* gadolinium-enhancing lesion, *GFAP* glial fibrillary acidic protein, *FDR* false discovery rate, *n.s.* not significant

Potential clinical and imaging predictors of aggressive MS disease course computed with the biomarkers sGFAP and cGFAP as a dependent variable, while controlling for the baseline characteristics. Before analysis, serum and CSF parameters were log-transformed. Results were corrected for false-discovery rate (FDR) using the Benjamini–Hochberg procedure. Adjusted and corrected for multiple comparisons *q* values for each predictor were calculated and significance level was set at *q* < 0.05. Presence of “2 or more Gd+ lesions” was defined for two or more Gd+ lesions on brain and/or spinal imaging

### Characterization of pwMS with elevated sNfL and sGFAP levels

To better understand which characteristics distinguish patients with the highest sNfL and sGFAP concentrations from the other patients, we extracted the data for the subgroups with elevated sNfL > 10 pg/ml, sNfL in the highest quartile (>17.5 pg/ml), sGFAP in the highest quartile (>109 pg/ml), and the combined elevation of both biomarkers. Tables 5 and 6 present the respective baseline characteristics as well as the ECTRIMS criteria for an aggressive MS disease course compared to the whole sample.

**Table 5:** Characterization of patients with serum NfL in the highest quartile

	All patients *n* = 99		4th quartile serum NfL, >17.5 pg/ml *n* = 24		4th quartile serum GFAP, >109 pg/ml *n* = 24		Serum NFL > 10.0 pg/ml *n* = 55		Combined 4th quartile serum NfL and 4th quartile serum GFAP *n* = 9	
Age (years), mean (SD)	34.0 (± 9.5)		33.9 (± 10.3)		33.9 (± 10.3)		33.9 (± 9.3)		34.1 (± 11.5)	
Female, *n* (%)	77	77.7%	21	87.5%	21	87.5%	44	80%	8	88.9%
Male, *n* (%)	22	22.2%	3	12.5%	3	12.5%	11	20%	1	11.1%
RRMS, *n* (%)	77	77.7%	24	100%	22	91.7%	47	85.5%	9	100%
CIS, *n* (%)	22	22.2%	0	0.0%	2	8.3%	8	14.5%	0	0.0%
BMI (kg/m^2^), mean (SD)	25.0 (± 5.2)		25.0 (± 6.1)		25.0 (± 6.1)		24.6 (± 5.6)		22.1 (± 4.3)	
EDSS, mean (SD)	1.9 (± 1.2)		2.1 (± 1.3)		2.1 (± 1.3)		2.0 (± 1.2)		2.5 (± 1.4)	
Gadolinium-enhancing T1-weighted lesions on MRI, *n* (%)										
Yes	58	58.5%	21	87.5%	16	66.7%	37	67.3	7	77.8%
No	35	35.4%	2	8.3%	7	29.2%	15	27.3	1	11.1%
Gd not administered	6	6.1%	1	4.2%	1	4.2%	3	5.5	1	11.1%
With acute relapse, *n* (%)	86	86.9%	22	91.7%	24	100%	50	90.1%	9	100%
Blood-CSF barrier dysfunction, *n* (%)	19	19.2%	6	25.0%	3	12.5%	10	18.2%	7	22.2%
CSF leucocytes count (/mm^3^), mean (SD)	9.9 (± 12.6)		12.7 (± 8.7)		12.8 (± 8.7)		10.2 (± 8.8)		13.6 (± 11.7)	
Intrathecal synthesis of IgG, *n* (%)	60	60.6%	19	79.2%	17	70.8	34	61.8%	7	77.8%
Positive oligoclonal bands, *n* (%)	90	90.9%	24	100%	24	100%	51	92.3%	9	100%
CSF/blood albumin quotient, mean (SD)	5.0 (± 2.1)		5.3 (± 2.0)		5.3 (± 2.0)		5.1 (± 2.1)		5.5 (± 2.0)	

*RRMS* relapsing–remitting multiple sclerosis, *CIS* clinically isolated syndrome, *EDSS* Expanded Disability Status Scale, *Gd* gadolinium, *MRI* magnetic resonance imaging, *CSF* cerebrospinal fluid, *IgG* immunoglobulin G, *NfL* neurofilament light, *GFAP* glial fibrillary acidic protein, *SD* standard deviation

Demographic, clinical and laboratory characteristics of patients with serum NfL in the highest quartile (above 17.5 pg/nL) in comparison to all patients

**Table 6:** Clinicoradiological markers of an aggressive MS disease course in patients with elevated sNfl and/or sGFAP

	All patients *n* = 99		4th quartile serum NfL, > 17.5 pg/ml *n* = 24		4th quartile serum GFAP, > 109 pg/ml *n* = 24		Serum NFL > 10.0 pg/ml *n* = 55		Combined 4th quartile serum NfL & 4th quartile serum GFAP *n* = 9	
	*n*	%	*n*	%	*n*	%	*n*	%	*n*	%
Motor symptoms at onset	15	16.3	5	21.7	7	29.2	13	23.6	1	11.1
Pyramidal signs in first year	21	21.9	8	33.3	12	50	17	30.9	4	44.4
Motor, cerebellar, cognitive or sphincter function	22	22.9	8	33.3	10	41.7	18	32.7	4	44.4
EDSS > 3.0 in first year	19	20.7	6	27.3	6	25	13	23.6	2	22.2
Severe relapses > 1 point EDSS	35	36.5	11	45.8	15	62.5	23	41.8	5	55.6
Poor recovery from first relapse	22	23.7	9	37.5	8	33.3	16	29.1	3	33.3
2 or more Gd+ lesions	35	38.5	20	83.3	12	50.0	28	50.9	6	66.7
At least one spinal cord lesion	41	45.1	11	45.8	14	58.3	24	43.6	4	44.4
Presence of infratentorial lesions	37	40.7	18	75.0	12	50.0	31	56.4	7	77.8
Presence of spinal > infratentorial lesions	27	29.9	4	16.7	7	29.2	12	21.8	1	11.1
More than 10 T2 lesions on brain MRI	17	17.7	5	20.8	3	12.5	10	18.2	0	0
More than 20 T2 lesions on brain MRI	25	26.0	13	54.2	10	41.7	19	34.5	6	66.7

*RRMS* relapsing–remitting multiple sclerosis, *CIS* clinically isolated syndrome, *EDSS* Expanded Disability Status Scale, *Gd+* *MRI* magnetic resonance imaging with gadolinium administration, *CSF* cerebrospinal fluid, *IgG* immunoglobulin G, *NfL* neurofilament light, *GFAP* glial fibrillary acidic protein, *SD* standard deviation

Possible predictors of aggressive MS disease course and the respective serum NfL and serum GFAP values of patients in the highest quartiles in comparison to all 99 patients. Patients with more than 20 T2 lesions are also included in “more than 10 T2 lesions on brain MRI”. Spinal MR imaging was available in 55 out of 99 patients, Gadolinium administration was available in 93 out of 99 patients

Baseline characteristics showed that an elevation of sNfL, and even more so the combination of both elevated sNfL and sGFAP were exclusively found in patients with RRMS and predominantly present in patients with Gd+ lesions and an acute relapse. Moreover, with exception of “Presence of spinal > infratentorial lesions”, all ECTRIMS criteria for an aggressive MS disease course were more commonly present if sNfL was elevated in the 4th quartile. This finding was predominantly present for the presence of 2 or more gadolinium-enhancing lesions (83.3% vs. 38.5% in total sample), presence of infratentorial lesions on brain MRI (75.0% vs. 40.7%), and for more than 20 T2 lesions on brain MRI (54.2% vs. 26.0%). An increase of sNfL above the cutoff value of 10 pg/ml, as previously introduced in the literature [37], showed a similar tendency, though weaker discrimination. With the exception of motor symptoms at disease onset and at least one spinal cord lesion, the combination of increased sNfL and cGFAP selected patients with more risk factors for an aggressive MS disease course than increased sNfL alone.

## Discussion

In patients with aggressive MS, early access to high efficacy therapies can slow down the disease progression and prevent severe long-term disability accumulation. Moreover, there is evidence that the therapeutic window is narrow, suggesting early treatment with high efficacy therapies is preferable over escalation strategies in patients with aggressive MS disease courses [19, 40]. Therefore, the early identification of those patients at risk is vital to reduce disability progression and preserve life quality. To date, there is no standardized definition for aggressive MS, making the early identification of patients difficult. The ECTRIMS Consensus group discussed risk factors characterizing aggressive MS disease courses but it remained unclear to which extent these clinical predictors of aggressive MS can be matched to biochemical measures of neuroaxonal and astroglial damage.

In recent years, the diagnostic and prognostic utility of fluid biomarkers, especially NfL, has been demonstrated in MS patients. There is strong evidence that patients with higher NfL concentrations are at risk for long-term disability [41, 42] with worse clinical and MRI outcome [43], developing a higher rate of brain atrophy in the following 2 years compared to patients with lower values [44, 45]. Extremely high sNfL levels indicate subclinical disease activity and could drive therapeutic decision making in individual patients [46]. Furthermore, NfL can help in selecting patients with a high probability of a progressive disease course [42, 47, 48]. NfL in serum after disease onset was also shown to reliably predict NEDA-3 status [48, 49] as well as T1-hypointense lesions over a 6-year follow-up period [48]. Moreover, NfL can be relevant for therapeutic response monitoring: sNfL concentrations of MS patients treated with high efficacy therapies as Natalizumab or Fingolimod goes back to the level of healthy controls, significantly exceeding the amount of decrease in patients treated with platform therapies [50–52]. In addition to sNfL, also sGFAP has recently shown its complementary potential as a prognostic biomarker for future disease progression and accelerated gray-matter brain volume loss in pwMS, especially when combing the elevation of z-scores of both, sNfL and sGFAP [38]. GFAP was shown also to be higher in NMOSD compared to MS or MOGAD with increasing levels in CSF and serum during acute relapses being associated with disability. However, astrocytic damage is more severe and occurs earlier during acute relapses in NMOSD compared to MS, therefore leading to higher concentrations of GFAP in CSF and serum and making assessment easier [53]. Possibly, this could be one potential explanation of the negative findings from our study with regard to GFAP.

How well serum/CSF concentrations of NfL and GFAP overlap with the risk factors of aggressive MS proposed by the ECTRIMS Consensus group [2] is undetermined. In this study, we aimed to match objective measures of neuroaxonal and astroglial damage to proposed clinical and paraclinical characteristics of patients at risk for an especially severe disease burden. This would allow to describe a biopathological valid “fingerprint” of aggressive disease course criteria.

We demonstrated that the presence of 2 or more gadolinium-enhancing lesions as well as the presence of infratentorial lesions is strongly associated with higher NfL concentrations as marker for neuroaxonal damage in serum as well as CSF after controlling for covariates. These findings are in line with the evidence from other studies suggesting that these MRI criteria provide relevant insight into the individual prognosis already in early disease stages and are predictors for essential disease activity, disease progression and accumulation of long-term disability, as recently reviewed in Hoffmann et al. [54].

However, no correlations with disability captured by EDSS was found, which is in line with the literature [47, 55–58], though the reports are inconclusive [55, 57, 59–61]. Still, none of the clinical risk factors of aggressive MS correlated with NfL and GFAP as markers of neuroaxonal and astroglial damage. In this study, only imaging criteria, but not clinical characteristics were significantly associated with serum and CSF NfL concentrations in pwMS. Several explanations for these results have to be considered.

First, the higher interrater variability of clinical characteristics with better objectivity of imaging findings in contrast to clinical characteristics needs to be considered. Especially, the individual EDSS rating can bear significant interrater variability [62], whereby recording of cognitive or vegetative deficits frequently is only rudimentarily performed.

Another explanation, however, could be that clinical criteria are less appropriate for assessing the extent of neuroaxonal damage. If they affect significant regions such as the corona radiata or brainstem, even small cerebral lesions can result in pronounced clinical symptoms, with less NfL being released than in a cumulatively high lesion load with only subtle clinical symptoms. On the other hand, patients with a higher lesion load might still have enough cerebral reserves available allowing them to compensate for the corresponding neuroaxonal damage and exhibit only low levels of neurological deficits. This “clinical-imaging mismatch” or “clinico-radiological paradox” [63] might be a possible explanation of our findings. Additional studies with larger patient cohorts are needed for further evaluation.

Fluid biomarkers as NfL or GFAP for assessment of aggressive MS have some advantages over MRI marker of disease activity. It has to be considered that MRI parameters (e.g., lesion load; exception: Gd+ lesions) are mostly results of previous disease activity, while fluid biomarkers as NfL reflect ongoing disease activity and are elevated up to 6 years before the first clinical manifestation [64, 65]. Moreover, assessment of brain atrophy as well as cortical lesions is not widely available and therefore easily accessible objective biomarkers for evaluation of the risk of aggressive MS are warranted. The limited correlation between clinical disability and MRI parameters (clinico-radiological paradox) [63, 66, 67] indicates that treatment decisions based solely on clinical presentation could be insufficient [66]. In addition, only brain MRI is performed regularly for disease monitoring, but the assessment of spinal cord MRI is much less frequent. Here, NfL could provide additional diagnostic information as it reflects actual neuroaxonal damage in the whole CNS and thus takes into consideration also disease activity in the spinal cord [65], though it has to be considered that no differentiation between neurodegenerative and inflammatory processes leading to an increase of the NfL level is possible.

Interestingly, the characterization of selected pwMS with elevated levels of sNfL showed a similar, moderately increased presence of risk factors for an aggressive disease course in patients with a cutoff > 10 pg/ml and patients with a cutoff > 17.5 pg/ml sNfL (4th quartile in study sample) when compared to the whole study sample. This underlines the value of sNfL as a driver and complementor of treatment decisions in MS, especially for ambiguous cases. However, the findings indicate a more linear relationship for the radiological items “presence of 2 or more Gd+ lesions”, “presence of infratentorial lesions”, and “more than 20 T2 lesions on brain MRI”, which highlights the strength of the biomarker sNfL in correlating objective, structural CNS injury (neuroaxonal injury) with increasing blood levels. This finding goes well in agreement with the results of the primary analysis, that showed best correlations for sNfL and cNfL with objective measures of structural CNS injury. However, we did not find any correlation of GFAP with clinicoradiological characteristics of aggressive MS though studies revealed release of GFAP in CSF and serum through CNS damage [68]. Still, some studies reported that GFAP break down product concentrations are higher in serum than concentration of full length GFAP, therefore making it more sensitive to detect astrocytic damage [53, 69]. While it is not entirely clear whether reactive astrocytosis might contribute majorly to increased serum GFAP levels in MS [70], there are several studies suggesting direct associations between astrocyte damage and elevated GFAP serum concentrations [71]. More precisely, evidence from studies on traumatic brain injury and stroke show increasing GFAP blood levels already few hours after the corresponding damage, thus suggesting GFAP release due to damaged astrocytes [38, 72]. Furthermore, in MS, studies revealed higher EDSS scores in patients with higher GFAP levels, supporting the hypothesis of increasing GFAP due do astrocytic damage [38].

Our study is not without limitations. MRI was performed in clinical routine; therefore, no information on brain atrophy or cortical lesions was applicable, which is why no correlation of brain or lesion volume with NfL or GFAP could be evaluated. However, as MRI was assessed in clinical routine, especially spinal MRI was not available in all patients. Moreover, 4 patients already were treated with high efficacy drugs, therefore, GFAP and NfL level could be decreased in those patients compared to patients without treatment at time of measurement, influencing the results. However, the secondary analysis focusing only on treatment-naïve patients confirmed our findings. Furthermore, no longitudinal follow-up was performed, though recent studies revealed that NfL levels already increase before clinical relapse and can progression independent of relapse activity [73]. It could be also argued that the temporary freezing at −20 °C could affect our findings. However, the transfer between the −20 and −80 °C freezer was done within few minutes while always ensuring that no thawing occurs. Furthermore, several studies suggested that NfL concentration is especially robust to factors such as delayed freezing (up to 8 days) and repetitive thawing (up to 4 thaws) [28, 74–76]. Several works have been already published following this algorithm [28–33].

## Conclusions

The findings of the current study indicate that MRI parameters but not clinical parameters considered risk factors of an aggressive disease course in pwMS correlate strongly with NfL as a marker of neuroaxonal injury in serum and CSF. No such correlation was apparent for GFAP as a marker of astroglial injury. MRI findings should be given appropriate weight when deciding on the type of disease-modifying therapy.

## Data Availability

The data that support the findings of this study are available from the corresponding author upon reasonable request.
